# Radiation exposure, young age, and female gender are associated with high prevalence of *RET/PTC1* and *RET/PTC3* in papillary thyroid cancer: a meta-analysis

**DOI:** 10.18632/oncotarget.7574

**Published:** 2016-02-23

**Authors:** Xuan Su, Zhaoqu Li, Caiyun He, Weichao Chen, Xiaoyan Fu, Ankui Yang

**Affiliations:** ^1^ Department of Head and Neck, Sun Yat-sen University Cancer Center, State Key Laboratory of Oncology in South China, Collaborative Innovation Center for Cancer Medicine, Guangzhou, China; ^2^ Department of Molecular Diagnostics, Sun Yat-Sen University Cancer Center, State Key Laboratory of Oncology in South China, Collaborative Innovation Center for Cancer Medicine, Guangzhou, China

**Keywords:** RET/PTC, papillary thyroid cancer, radiation, biomarker

## Abstract

**Background:**

*RET/PTC* rearrangements have been identified as a specific genetic event in papillary thyroid cancer (PTC). We conducted this meta-analysis to identify an enriched population who were more likely to occur *RET/PTC* fusion genes.

**Methods:**

All relevant studies in the PubMed, Web of Science, and Embase databases were searched up to June 2015. The studies found were screened according to our inclusion and exclusion criteria. All analyses were performed using STATA software.

**Results:**

Eventually, 38 eligible studies comprising 2395 participants were included. Overall analysis indicated that radiation exposure contributed to increased *RET/PTC* risk (OR = 2.82; 95%CI: 1.38–5.78, P = 0.005). Stratified analysis according to *RET/PTC* subtype and geographical area showed that this association was restricted to the *RET/PTC3* subtype (OR = 8.30, 95%CI: 4.32–15.96, P < 0.001) in the Western population. In addition, age < 18 years, i.e., young age, was associated with higher prevalence of *RET/PTC3* (OR = 2.03, 95%CI: 1.14–3.62, P = 0.017), especially in the radiation-exposure subpopulation (OR = 2.35, 95%CI: 1.01–5.49, P = 0.048). The association between female gender and *RET/PTC1* risk was more significant in the PTC patients without radiation exposure (OR = 1.69, 95%CI: 1.04–2.74, P = 0.034).

**Conclusion:**

Both radiation exposure and young age are associated with increased risk of *RET/PTC3* and that female gender is associated with higher prevalence of *RET/PTC1* in the subpopulation without radiation exposure. The *RET/PTC* status in combination with radiation exposure, age, and sex should be considered in the differential diagnosis of suspicious PTC.

## INTRODUCTION

Rearrangement involving the *RET* proto-oncogene, referred to *RET/PTC* (the rearranged during transfection/papillary thyroid carcinoma tyrosine kinase) fusion genes, is one of the best-known mutations in papillary thyroid carcinoma (PTC) [1]. *RET/PTC* is identified as a specific genetic event in patients with PTC, which forms the basis of differential diagnosis and novel therapeutic approaches to this disease [2]. However, the prevalence rate of the major *RET/PTC* subtypes in different ethnicities and their correlation with the clinicopathologic features of PTC remains controversial and as yet are not routinely investigated in clinical practice.

The receptor tyrosine kinase RET plays a critical role in cell differentiation and proliferation, which is required for normal development of several tissues, especially in early embryogenesis [3]. In 1985, Takahashi and colleagues initially reported *RET* as a proto-oncogene that can be activated by interchromosomal rearrangement [4]. Subsequent studies demonstrated more than a dozen different forms of *RET* rearrangement, of which *RET/PTC1* and *RET/PTC3* are the most common, resulting from the fusion of *RET* with *H4* and of *RET* with *RFG/ELE1* respectively [5]. However, their prevalence rates in PTC exhibit significant geographic variation, ranging from 0% to 86.7% among studies [6, 7]. As risk factors such as sex, age, and radiation exposure are related to PTC pathogenesis, scientists have focused on searching for the factors that increase *RET/PTC*rearrangement risk. Accordingly, several studies have detected *RET/PTC* rearrangements more frequently in PTC in children than in adults [8, 9]. A relatively high prevalence of *RET/PTC* rearrangements was reported in radiation-induced PTC [10]. In addition, ethnicity and demographic characteristics may also influence the frequency of *RET/PTC* rearrangement [11-13]. To date, numerous relevant studies have been published but with divergent results.

In this study, we aimed to identify an enriched population who were more likely to occur RET/PTC1 and RET/PTC3 fusion genes and to provide more useful information on candidate selection for PTC prevention, diagnosis, and treatment. Therefore, we performed this meta-analysis to investigate the association between the presence/absence of *RET/PTC1* or *RET/PTC3* and radiation exposure, sex, age, and ethnicity.

## RESULTS

### Basic characteristics of enrolled studies

**Figure 1:**
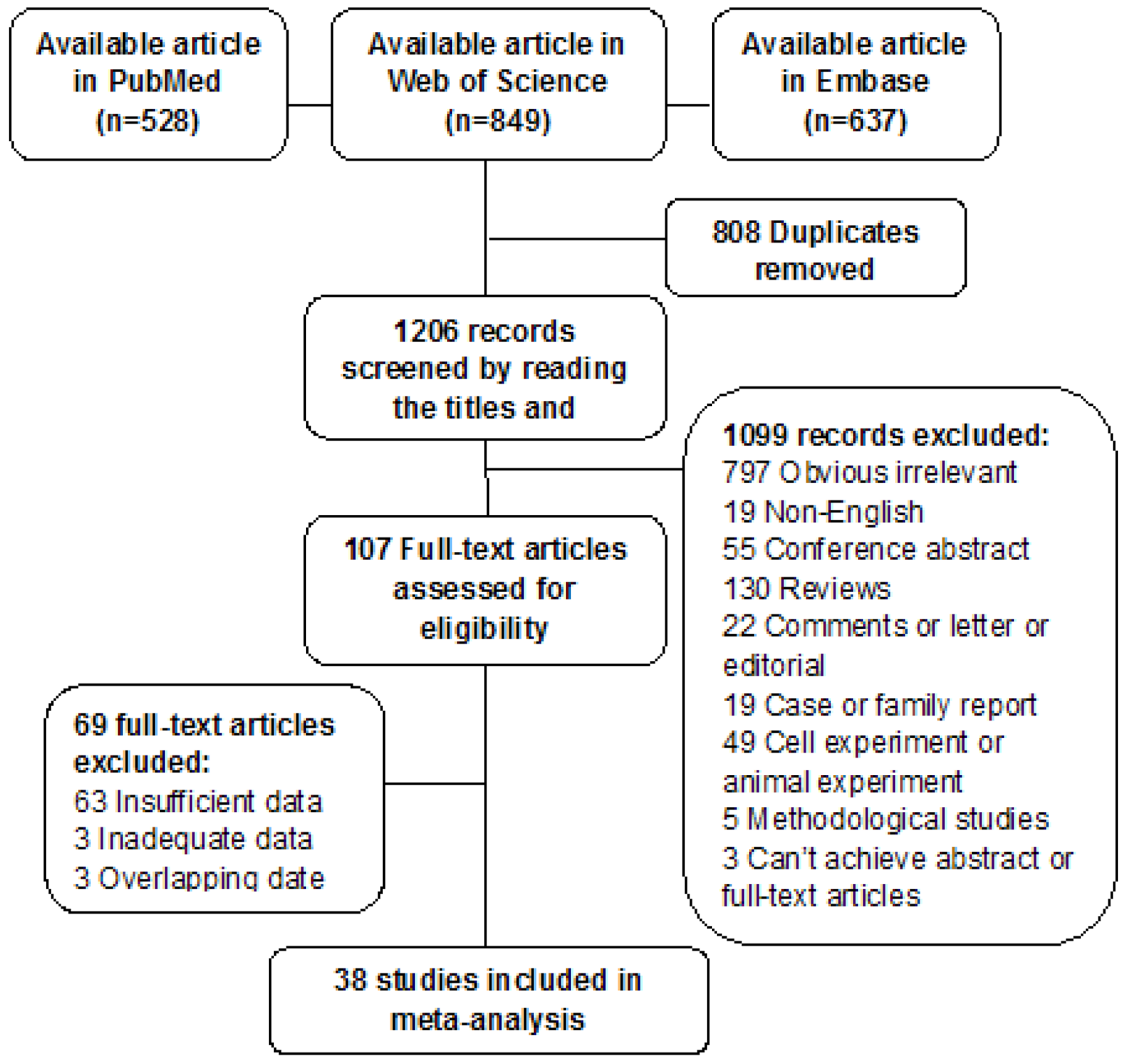
Flowchart of literature search and selection of studies

The article selection flowchart is depicted in Figure 1. A total 2014 records were obtained by searching the PubMed, Web of Science, and Embase databases. After removing duplicates, we found 1206 potentially relevant records. By reviewing titles, abstracts, and full texts according to the inclusion and exclusion criteria, 1168 articles were excluded because they were not relevant, involved in vitro or animal experiments, were reviews or meeting abstracts, contained data covered by other studies, had no raw data, etc. Eventually, 38 full-text articles, which consisted of 2395 PTC cases, met our inclusion criteria and were included in the final meta-analysis [6-10, 14-47].

The main characteristics of the studies included in this meta-analysis are summarized in Table 1. The status of the *RET/PTC* fusion gene in the original studies were detected by PCR combined with reverse transcription (RT), Southern Blot or fluorescence in situ hybridization (FISH). The included studies involved populations from different geographical regions, namely Asia, Europe, and America; therefore, we divided the studies into Asian and Western subgroups.

**Table 1:** Characteristics of studies included in the meta-analysis

First author	Year of publication	Ethnicity	Region	Method of detection	Radiation	No.of patients
Nikiforov et al	1997	Belarussian, Los Angeles, Cincinnati	Western	RT-PCR	Mixed	55
Bounacer et al	1997	French	Western	RT-PCR	Mixed	39
Motomura et al	1998	Japanese	Asian	RT-PCR	Non-radiation	21
Smida et al	1999	Belarussian, German	Western	RT-PCR	Mixed	83
Rabes et al	2000	Belarus, Russia, Ukrainian	Western	PCR	Radiation	191
Elisei et al	2001	Belarus, Itaian	Western	PCR	Mixed	89
Puxeddu et al	2003	Italian	Western	RT-PCR, Southern Blot	Non-radiation	48
Rhoden et al	2004	American	Western	RT-PCR	NM	25
Nakazawa et al	2005	Japanese	Asian	RT-PCR	Non-radiation	169
Brzezianska et al	2006	Polish	Western	RT-PCR	NM	33
Hamatani et al	2008	Japanese	Asian	RT-PCR	Mixed	71
Tuttle et al	2008	Russian	Western	RT-PCR	Radiation	76
Lam et al	2002	China, Hong Kong	Asian	RT-PCR	Non-radiation	21
Detours et al	2005	Ukrainian	Western	RT-PCR	Mixed	20
Lima et al	2004	Ukrainian	Western	RT-PCR	Mixed	34
Penko et al	2005	American	Western	PCR	Mixed	13
Romei et al	2008	Italian	Western	RT-PCR	Non-radiation	70
Hieber et al	2011	Ukrainian	Western	FISH	Radiation	22
Guerra et al	2014	Italian	Western	RT-PCR	NM	72
Zou et al	2014	Saudi Arabian	Asian	RT-PCR	NM	88
Chung et al	1999	Korean	Asian	RT-PCR	Non-radiation	31
Powell et al	2005	Ukrainian	Western	PCR	Mixed	35
Unger et al	2004	Ukrainian	Western	FISH	Radiation	29
Wang et al	2008	Chinese	Asian	RT-PCR	Non-radiation	126
Nikiforova et al	2004	Belorussian, Ukrainian	Western	PCR	Mixed	137
Basolo et al	2002	Italian	Western	RT-PCR	NM	91
Rao et al	2014	Indian	Asian	RT-PCR	Non-radiation	30
Collins et al	2002	American	Western	IHC	Mixed	64
Chung et al	2004	Korean	Asian	RT-PCR+IHC	Non-radiation	22
Unger et al	2006	Ukrainian	Western	FISH	Radiation	13
Sadetzki et al	2004	Israelis	Asian	RT-PCR	Mixed	49
Smyth et al	2005	Irish	Western	Taqman	NM	34
Learoyd et al	1998	Australian, Swedish	Western	RT-PCR	Mixed	50
Nakazawa et al	2009	Japanese	Asian	FISH+RT-PCR	Non-radiation	14
Di Cristofaro et al	2005	Ukrainian, French	Western	RT-PCR	Mixed	50
Erdogan	2008	Turkish	Asian	RT-PCR	Non-radiation	101
Fenton et al	2000	American	Western	PCR	Non-radiation	33
Guerra et al	2011	Italian	Western	RT-PCR	NM	50
Stanojevic et al	2011	Serbian	Western	PCR	Non-radiation	266

We also summarized the positive rates of *RET/PTC* from each original study (Table 2). The overall prevalence of *RET/PTC* was relatively higher in the Western populations (42.19%) than in the Asian populations (36.73%). A similar tendency was observed for the *RET/PTC1* subtype, whereas the Asian populations demonstrated a higher positive rate for the *RET/PTC3* subtype (Asian vs. Western populations: 26.50% vs. 17.05%). In the Asian studies, the positive rates of *RET/PTC3* in the studies by Lam et al. [16] and Rao et al. [7] were up to 85.71% and 86.67%, respectively, while another six studies reported a much lower incidence of *RET/PTC3* that ranged from 0% to 20.79% [9, 14, 20-23].

**Table 2:** Positive rates of *RET/PTC1* and *RET/PTC3* in each original study

First author	Year of publication	No. of PTC cases	Freq. of RET/PTC1 and 3	RET/PTC1 and 3(%)	Freq. of RET/PTC1	RET/PTC1(%)	Freq. of RET/PTC3	RET/PTC3(%)
**For Asian studies**				**36.73%**		**21.06%**		**26.50%**
Motomura et al	1998	21	7	33.33%	5	23.81%	2	9.52%
Nakazawa et al	2005	169	48	28.40%	43	25.44%	8	4.73%
Hamatani et al	2008	71	12	16.90%				
Lam et al	2002	21	18	85.71%			18	85.71%
Zou et al	2014	88	12	13.64%	12	13.64%		
Chung et al	1999	31	4	12.90%				
Wang et al	2008	126	18	14.29%				
Rao et al	2014	30	26	86.67%	0	0.00%	26	86.67%
Chung et al	2004	22	2	9.09%	1	4.55%	1	4.55%
Sadetzki et al	2004	49	22	44.90%	20	40.82%	0	0.00%
Nakazawa et al	2009	14	4	28.57%	4	28.57%	0	0.00%
Erdogan	2008	101	67	66.34%	32	31.68%	21	20.79%
**For Western studies**				**42.19%**		**25.25%**		**17.05%**
Nikiforov et al	1997	55	40	72.73%	14	25.45%	25	45.45%
Bounacer et al	1997	39	18	46.15%	15	38.46%	5	12.82%
Smida et al	1999	83	39	46.99%	26	31.33%	13	15.66%
Rabes et al	2000	191	86	45.03%	48	25.13%	38	19.90%
Elisei et al	2001	89	40	44.94%	18	20.22%	26	29.21%
Puxeddu et al	2003	48	13	27.08%	8	16.67%	5	10.42%
Rhoden et al	2004	25	18	72.00%	18	72.00%	5	20.00%
Brzezianska et al	2006	33	7	21.21%				
Tuttle et al	2008	76	13	17.11%	11	14.47%	5	6.58%
Detours et al	2005	20	7	35.00%	1	5.00%	2	10.00%
Lima et al	2004	34	14	41.18%				
Penko et al	2005	13	7	53.85%	5	38.46%	2	15.38%
Romei et al	2008				13	18.57%	12	17.14%
Hieber et al	2011	22	17	77.27%				
Guerra et al	2014	72	12	16.67%				
Powell et al	2005	35	16	45.71%				
Unger et al	2004	29	5	17.24%	2	6.90%	3	10.34%
Nikiforova et al	2004	137	48	35.04%	16	11.68%	32	23.36%
Basolo et al	2002	91	28	30.77%	6	6.59%	22	24.18%
Collins et al	2002	64	44	68.75%				
Unger et al	2006	13	10	76.92%				
Smyth et al	2005	34	13	38.24%	10	29.41%	3	8.82%
Learoyd et al	1998	50	4	8.00%	4	8.00%	0	0.00%
Di Cristofaro et al	2005	50	30	60.00%	26	52.00%	13	26.00%
Fenton et al	2000	33	14	42.42%	11	33.33%	3	9.09%
Guerra et al	2011	50	18	36.00%				
Stanojevic et al	2011	266	55	20.68%	42	15.79%	13	4.89%

### Association between radiation exposure and RET/PTC fusion genes

**Figure 2:**
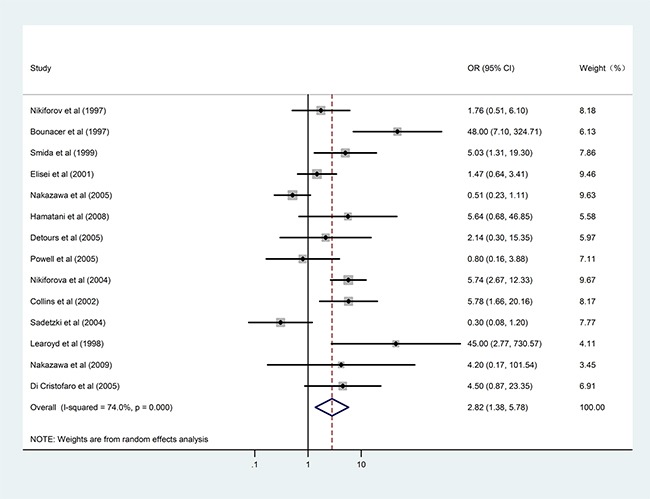
Results of the association between *RET/PTC1* and *RET/PTC3* fusion genes and radiation exposure in patients with PTC

As radiation exposure is the best-known risk factor for PTC, we initially investigated the effect of radiation on *RET/PTC* rearrangement (Table 3). Fourteen studies investigated the distribution difference of radiation exposure between *RET/PTC*-positive and -negative patients with PTC. When the *RET/PTC1* and *RET/PTC3* subtypes were combined, radiation exposure conferred increased overall risk for *RET/PTC* development (OR = 2.82, 95%CI: 1.38–5.78; P = 0.005, Figure 2) and there was moderate heterogeneity (I^2^ = 74%, P_het_ < 0.001). Stratified analysis according to geographical region decreased the heterogeneity slightly, and increased risk for *RET/PTC* rearrangement persisted in the Western subpopulation, demonstrating an increased OR of 3.97 (95%CI: 2.03–7.75; P < 0.001).

**Table 3:** Meta-analysis results for association between *RET/PTC* fusion genes and radiation exposure in patients with PTC

Radiation exposure vs. non-radiation exposure	No. of studies	No. of cases/controls	OR(95%CI)	P value	Model	I^2^	Phet^a^
**For RET/PTC1 and 3**							
All	14	537/388	**2.82(1.38,5.78)**	**0.005**	Random	74%	<0.001
Region							
Asian	4	232/71	0.88(0.26,2.93)	0.833	Random	56%	0.077
Western	10	305/317	**3.97(2.03,7.75)**	**<0.001**	Random	59%	<0.001
**For RET/PTC1**							
All	9	285/287	1.86(0.66, 5.28)	0.243	Random	76.00%	<0.001
Region							
Asian	1	37/12	0.24(0.06,0.96)	0.043	Random	/	/
Western	8	248/275	2.46(0.83,7.27)	0.104	Random	74.10%	<0.001
**For RET/PTC3**							
All^b^	8	243/240	**8.30(4.32,15.96)**	**<0.001**	Fixed	0.00%	0.980
Region							
Asian	/	/	/	/	/	/	/
Western	8	243/240	**8.30(4.32,15.96)**	**<0.001**	Fixed	0.00%	0.980

a P-value for heterogeneity test;

b Data from Sadetzki et al. [21] and Learoyd et al. [6] showed that the *RET/PTC3* gene prevalence was 100% in both the groups with and without radiation exposure and that the OR and standard error could not be estimated; therefore, these studies were excluded. The statistically significant results are highlighted in bold.

**Figure 3:**
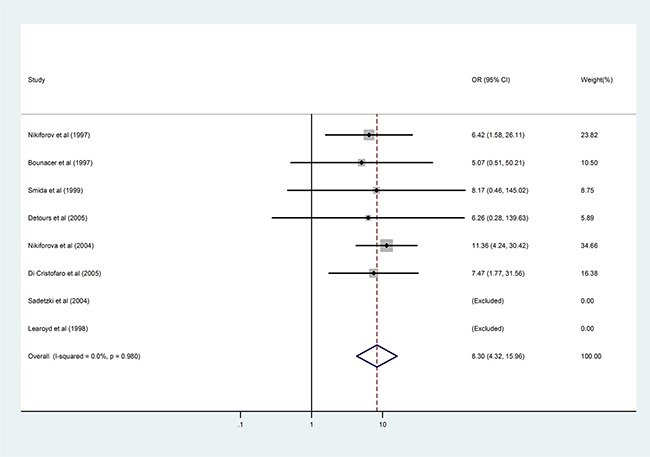
Results of the association between *RET/PTC3* fusion gene and radiation exposure in patients with PTC

When the *RET/PTC1* and *RET/PTC3* subtypes were considered separately, radiation exposure conferred significantly higher risk for *RET/PTC3* rearrangement (OR = 8.30, 95%CI: 4.32–15.96; P < 0.001, Figure 3) but not for *RET/PTC1* rearrangement. This association was only evident in the Western subpopulation. Separate pooled analysis for the *RET/PTC1* and *RET/PTC3* subtypes was not performed for the Asian subpopulation because three original studies involving this population investigated the combined status of *RET/PTC1* and *RET/PTC3*, and the data could not be extracted separately [9, 15, 22]. Only one study with a small sample in an Asian country reported the *RET/PTC1* and *RET/PTC3* fusion gene data separately [21]. There was significant inter-study heterogeneity in the *RET/PTC1* analysis but not in the *RET/PTC3* analysis.

### Association between RET/PTC fusion genes and age

**Figure 4:**
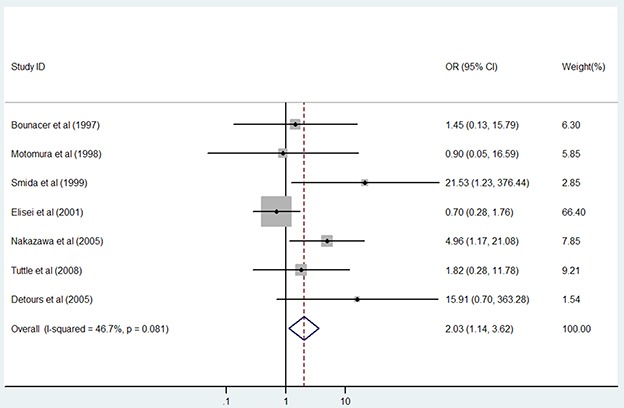
Results of the association between *RET/PTC3* fusion gene and young age in patients with PTC

Different age effects have been observed in the development of PTC, and we therefore explored whether young age affected the penetrance of the*RET/PTC* fusion genes in children and adolescents (Table 4). In this meta-analysis, age < 18 years was considered young, i.e., children and adolescents. In the combined analysis of the *RET/PTC1* and *RET/PTC3* subtypes, no association was observed between age and *RET/PTC* rearrangement. In the separate analysis of the *RET/PTC1* and *RET/PTC3* subtypes, young people had nearly two-fold greater risk for *RET/PTC3* rearrangement (Figure 4) but penetrance of the *RET/PTC1* fusion gene was not affected. When radiation exposure was also considered, young people in the subpopulation with radiation exposure had higher risk for developing *RET/PTC3* rearrangement as compared to adults with radiation exposure. These positive associations were performed in a fixed-effects model and had slight inter-study heterogeneity (all Phet > 0.10).

**Table 4:** Meta-analysis results for association between *RET/PTC* fusion genes and age in patients with PTC

Young people vs. adult	No. of studies	No. of cases/controls	OR(95%CI)	P value	Model	I^2^	Phet^a^
**For RET/PTC1 and 3**							
All	8	187/332	1.10(0.56,2.16)	0.783	Random	51.50%	0.044
Region							
Asian	2	41/149	1.76(0.84,3.69)	0.133	Fixed	4.30%	0.307
Western	6	146/183	0.98(0.41,2.31)	0.956	Random	54.50%	0.052
Radiation							
Radiation exposure	6	116/122	0.88(0.48,1.62)	0.682	Fixed	41.50%	0.129
Non-radiation exposure	5	71/200	1.46(0.81,2.65)	0.212	Fixed	33.90%	0.195
**For RET/PTC1**							
All	6	172/286	0.98(0.60,1.58)	0.921	Fixed	7.20%	0.370
Region							
Asian	2	41/149	1.33(0.61,2.91)	0.476	Fixed	0.00%	0.466
Western	4	131/137	0.81(0.44,1.50)	0.507	Fixed	21.40%	0.282
Radiation							
Radiation exposure	5	107/109	0.52(0.26,1.05)	0.070	Fixed	49.30%	0.096
Non-radiation exposure	5	71/200	1.47(0.76,2.86)	0.250	Fixed	0.00%	0.574
**For RET/PTC3**							
All	7	179/318	**2.03(1.14,3.62)**	**0.017**	Fixed	46.70%	0.081
Region							
Asian	2	42/148	3.23(0.87,12.00)	0.080	Fixed	7.20%	0.299
Western	5	137/170	1.84(0.97,3.50)	0.206	Random	50.1%%	0.091
Radiation							
Radiation exposure	5	107/109	**2.35(1.01,5.49)**	**0.048**	Fixed	0.00%	0.574
Non-radiation exposure	5	72/199	1.68(0.28,10.01)	0.570	Random	67.00%	0.028

a P-value for heterogeneity test. The statistically significant results are highlighted in bold.

### Association between RET/PTC fusion genes and sex

**Figure 5:**
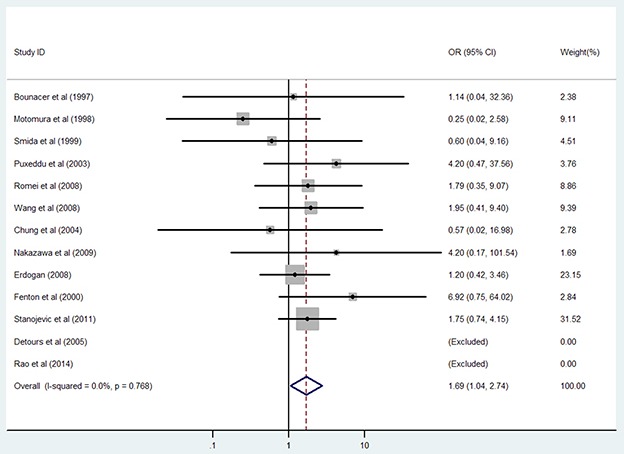
Results of the association between *RET/PTC1* fusion gene and female gender in PTC patients without radiation exposure

Females are more likely to develop PTC, therefore we investigated whether female gender increases the chance of *RET/PTC* rearrangement in patients with PTC. As suggested by the findings in Table 5, sex was not statistically associated with *RET/PTC* status in the combined analysis. In subgroup analysis, a pooled analysis of 13 studies showed that female gender was associated with *RET/PTC1* development in the subpopulation without radiation exposure. Female patients had 1.69-fold greater risk for *RET/PTC1* rearrangement than male patients did (95%CI: 1.04–2.74; P = 0.034, Figure 5). However, female gender did not appear to play a role in *RET/PTC3* rearrangement (all, P > 0.05). Only slight inter-study heterogeneity was observed in all of the above analyses (all Phet > 0.10).

**Table 5:** Meta-analysis results for association between *RET/PTC* fusion genes and sex in patients with PTC

Female vs. male	No. of studies	No. of cases/controls	OR(95%CI)	P value	Model	I2	Phet^a^
**For RET/PTC1 and 3**							
All	27	1211/474	1.04(0.81,1.33)	0.775	Fixed	0.00%	0.938
Region							
Asian	9	373/138	1.42(0.81,2.49)	0.216	Fixed	0.00%	0.754
Western	18	838/336	0.95(0.72,1.27)	0.747	Fixed	0.00%	0.934
Radiation							
Radiation exposure	11	369/174	0.94(0.63,1.41)	0.760	Fixed	0.00%	0.941
Non-radiation exposure	17	721/258	1.28(0.90,1.82)	0.171	Fixed	7.20%	0.370
**For RET/PTC1**							
All a	16	832/324	1.21(0.87,1.69)	0.256	Fixed	0.00%	0.857
Region							
Asian	5	213/62	0.98(0.48,2.01)	0.962	Fixed	0.00%	0.604
Western	11	619/261	1.28(0.88,1.87)	0.193	Fixed	0.00%	0.796
Radiation							
Radiation exposure	4	185/104	1.22(0.70,2.11)	0.482	Fixed	0.00%	0.892
Non-radiation exposure	13	581/192	**1.69(1.04,2.74)**	**0.034**	Fixed	0.00%	0.768
**For RET/PTC3**							
All,b	17	785/304	0.87(0.60,1.27)	0.466	Fixed	0.00%	0.625
Region							
Asian	5	155/40	1.54(0.59,3.99)	0.378	Fixed	0.00%	0.763
Western	11	619/261	0.77(0.51,1.17)	0.223	Fixed	0.00%	0.527
Radiation							
Radiation exposure	4	185/104	0.82(0.45,1.48)	0.504	Fixed	0.00%	0.903
Non-radiation exposure	11	570/186	1.06(0.60,1.87)	0.847	Fixed	0.00%	0.696

a P-value for heterogeneity test;

b Data from Rao et al. [7] and Detours et al. [31] showed that *RET/PTC1* gene prevalence was 100% in both female and male groups and that the OR and standard error could not be estimated; therefore, these studies were excluded. The statistically significant results are highlighted in bold.

### Heterogeneity testing and sensitivity analysis

The inter-study heterogeneities in each comparison are presented in Tables 3–5. Pooled analyses for assessing the effect of radiation exposure and young age on the combined status of the *RET/PTC1* and *RET/PTC3* fusion genes demonstrated moderate heterogeneity. To explore the source of heterogeneity, subgroup analyses based on ethnicity and *RET/PTC* subtype were performed. Heterogeneity was decreased in the subgroup analysis and may be partly explained by the different ethnicities and *RET/PTC* subtypes (Tables 3 and 5).

Sensitivity analysis was also conducted to assess the influence of individual studies on the overall risk of *RET/PTC* rearrangement by excluding any single study in turn and recalculating the pooled ORs and 95%CI. For the effect of radiation on *RET/PTC* rearrangement, Sadetzki et al. [21], Nakazawa et al. [22], and Bounacer et al. [24] reported greater differences in the risk estimates compared with other studies in the sensitivity analysis. Sensitivity analysis excluding the three studies generated a similar pooled OR of 3.30 (95%CI: 1.96–5.54, P < 0.001; I^2^= 32.1%, P_het_ = 0.142) among homogeneous studies. For the effect of young age on *RET/PTC* rearrangement, the outlier studies appeared to be the studies of Smida et al. (1999)[8] and Hieber et al. (2011)[35]. After removing the two studies, the heterogeneity was no longer significant (I^2^= 34.0%, P_het_ = 0.181), and similar estimates (OR = 1.10 vs. 1.53) were generated before and after these data were removed, indicating the relatively high stability of the results.

### Publication bias

Begg's test and Egger's test were performed to quantitatively evaluate the publication bias of the studies; the results are listed in Table 6. No significant publication bias was observed in all comparisons (all, P > 0.10).

**Table 6:** Analysis for publication bias

Variable	Begg's test		Egger's test	
	z value	P value^a^	t value	P value^a^
**For RET/PTC1 and 3**				
Radiation exposure vs. non-radiation exposure	0.93	0.352	1.34	0.205
Female vs. male	1.02	0.307	0.24	0.811
Children and adolecent vs. adult	−0.25	0.805	0.16	0.882
**For RET/PTC1**				
Radiation exposure vs. non-radiation exposure	1.25	0.211	1.71	0.132
Female vs. male	0.27	0.787	0.15	0.879
Children and adolecent vs. adult	−0.19	0.851	0.39	0.715
**For RET/PTC3**				
Radiation exposure vs. non-radiation exposure	−0.56	0.573	−1.75	0.156
Female vs. male	0.27	0.787	0.59	0.567
Children and adolecent vs. adult	0.45	0.652	1.92	0.113

a P value>0.1 was considered as no publication bias.

## DISCUSSION

Previous study results on the relationship between PTC-related risk factors and *RET/PTC* rearrangement were controversial. To our knowledge, this is the first meta-analysis evaluating the effect of radiation exposure, female gender, and young age on *RET/PTC* rearrangement. By performing the present meta-analysis, we found that radiation exposure contributed to increased risk of *RET/PTC* rearrangement, especially for the *RET/PTC3* subtype. Young age was also associated with higher prevalence of *RET/PTC3*, and this association was more significant in the subpopulation exposed to radiation. Our pooled estimate also demonstrated an association between female gender and higher prevalence of the *RET/PTC1* subtype in the subpopulation that had not been exposed to radiation. These results identify an enriched population of RET/PTC fusion genes in patients with PTC and provide novel insights into the utility of RET/PTC rearrangement in the differential diagnosis of suspicious PTC.

Exposure to ionizing radiation is a well-known risk factor for thyroid cancer, particularly for papillary carcinoma [48, 49]. Therefore, it is likely that radiation exposure may also be a causative factor for *RET/PTC* rearrangement. Our pooled estimates provide clear evidence that radiation exposure could be responsible for the difference in *RET/PTC3* prevalence between sporadic and radiation-associated tumors, whereas the rate of *RET/PTC1* prevalence was similar between the two groups. The corresponding pooled OR for *RET/PTC3* was up to 8.30, and this association was evident in the Western populations. However, we could not derive a negative or null association in the Asian population because of a lack of original studies from the Asian region. There are valid reasons to believe that there is a causative link between radiation exposure and *RET/PTC* rearrangements. For example, Nikiforov et al. reported much higher *RET/PTC3* prevalence in post-Chernobyl PTC than in subjects without radiation exposure [10]. In addition, *RET/PTC* rearrangement, predominantly *RET/PTC3*, in thyroid cells, can be induced by ionizing radiation [50]. This may be linked to the particular effectiveness of radiation in causing double-strand breaks, which would be the direct cause of RET rearrangement [50, 51]. This mechanism may partially explain the association between radiation exposure and *RET/PTC* rearrangement in thyroid cancer.

The data synthesis in the present meta-analysis also demonstrated increased risk of *RET/PTC3* in PTC in young people. Our observations further indicate that young patients who exposed to radiation have higher *RET/PTC3* risk than young patients who have not been exposed to radiation. Original studies have shown that *RET/PTC3* is more common in children and adolescents compared to adults [8, 9]. When the radiation exposure effect was considered in young people, *RET/PTC3* was indicated as the most common form of rearrangement in radiation-associated childhood PTC [8, 10, 25, 52]. As the thyroid is very sensitive to radiation, the thyroid of young people might be more vulnerable to radiation than that of adults, which may result in higher *RET/PTC* prevalence in young people [53]. Nevertheless, it is important to consider that the statistical power in each original study may be partly determined by the cutoff value of age, such as age at diagnosis and age at exposure to radiation. When the cutoff age varies, the issue of the impact of patient age on *RET/PTC* rearrangement remains inconclusive, which would require further validation. By setting a cutoff age of 18 years in this meta-analysis, the corresponding results may indicate the relatively low defense ability of children against pathogenic factors.

Concerning the impact of sex, we observed that the association between female gender and increased *RET/PTC1* risk was more significant in patients who had not been exposed to radiation. For unknown reasons, thyroid cancer is three times more prevalent in women than in men [54]. One possible explanation for this gender disparity is the hormonal differences between men and women. It has been documented that chromosome breaks and sister chromatid exchanges are elevated in women who are pregnant or taking oral contraceptives [55]. There is also evidence supporting the premise that *RET/PTC* is an estrogen-dependent gene required for breast cancer cell growth [56]. The above evidence suggests that some inherent differences render females more susceptible to *RET* rearrangements.

Nevertheless, this study has some limitations. First, although we included all available relevant articles in this meta-analysis, the sample sizes remain insufficiently large. Second, most studies in relation to the association between radiation exposure and *RET/PTC3* were from the Western countries, thus the generalizability of our conclusions is limited. In the future, more studies are needed to confirm this association in Asian regions. Third, only two common *RET/PTC* subtypes, *RET/PTC1* and *RET/PTC3*, were considered in this study, mainly due to the limitation of current laboratory techniques for simultaneously detecting all *RET/PTC* subtypes.

In conclusion, both radiation exposure and young age, i.e., age < 18 years, are associated with increased risk of *RET/PTC3* rearrangement. In addition, female gender is associated with higher prevalence of the *RET/PTC1* subtype in the subpopulation not exposed to radiation. We suggest that *RET/PTC* status in combination with radiation exposure, age, and sex should be considered when differential diagnoses are suggested for suspicious patients. Further large-scale studies concerning the relationship between radiation exposure and *RET/PTC* in the Asian population are required to confirm our meta-analysis results.

## MATERIALS AND METHODS

### Search strategy

We searched the PubMed, Web of Science, and Embase databases for all articles on the association between the *RET/PTC* fusion gene and PTC up to June 2015. The published date of available articles in this study was from 1959 to 2015. The keywords used for the search were “*RET/PTC*”, “*RET/PTC* fusion gene”, or “*RET/PTC* fusion oncoproteins” in combination with “Thyroid Cancer”, “Thyroid Carcinoma”, “Thyroid Neoplasms”, or “Thyroid Papillary Carcinoma”. The references of the articles acquired were also searched manually to broaden the search. When there was overlapping data, only the largest and most recent study was selected for this meta-analysis. If the data presented in an article were unclear, we contacted the author for specific raw data.

### Inclusion and exclusion criteria

Eligible studies had to meet the following criteria: (1) the association between the *RET/PTC* fusion gene and the clinicopathological features of patients with PTC was explored; (2) PTC diagnosis was made according to the pathology results; (3) studies were full-text articles; and (4) there was sufficient data for estimating an odds ratio (OR) with a 95% confidence interval (CI). The exclusion criteria were: (1) duplicate publication; (2) article was an abstract, comment, review, conference proceeding, or editorial; (3) insufficient data were reported; and (4) study was not in English or Chinese.

### Data extraction

The following items were collected: first name of first author; year of publication; population of study; number of enrolled patients; frequency of *RET/PTC* fusion gene; detection method; whether study subjects were children or adults; and clinicopathological features (sex, age, radiation history, and ethnicity). The above information was carefully extracted by two independent investigators (Xuan Su and Zhaoqu Li). If the two investigators could not reach a consensus, the result was reviewed by a third investigator (Caiyun He).

### Statistical analyses

The strength of the association between *RET/PTC* and radiation exposure, age, and sex was estimated by OR and 95%CI. Two-sided P-values were evaluated in this meta-analysis, and P < 0.05 was considered statistically significant. The chi-square–based Q test and I^2^ statistic were used to assess the statistical heterogeneity among studies. For the Q statistic, P < 0.10 was considered statistically significant for heterogeneity. When there was heterogeneity, a random-effects model based on the DerSimonian and Laird method was used to calculate the pooled OR of each study [57]; otherwise, a fixed-effects model based on the Mantel–Haenszel method was used [58]. Publication bias was examined using Begg's and Egger's tests [59, 60], where P < 0.10 was considered statistically significant. All analyses were performed using STATA 12.0. All tests were two-sided and the significance level was set at 0.05.
